# Regulated Cell Death Signaling Pathways and Marine Natural Products That Target Them

**DOI:** 10.3390/md17020076

**Published:** 2019-01-23

**Authors:** Esther A. Guzmán

**Affiliations:** Marine Biomedical and Biotechnology Research, Harbor Branch Oceanographic Institute at Florida Atlantic University, 5600 US 1 North, Fort Pierce, FL 34946, USA; eguzman9@fau.edu; Tel.: +772-242-2452

**Keywords:** marine natural products, regulated cell death, apoptosis, anoikis, paraptosis, necroptosis, ferroptosis, parthanatos, mitotic catastrophe, autophagy

## Abstract

Our understanding of cell death used to consist in necrosis, an unregulated form, and apoptosis, regulated cell death. That understanding expanded to acknowledge that apoptosis happens through the intrinsic or extrinsic pathways. Actually, many other regulated cell death processes exist, including necroptosis, a regulated form of necrosis, and autophagy-dependent cell death. We also understand that apoptosis occurs beyond the intrinsic and extrinsic pathways with caspase independent forms of apoptosis existing. Our knowledge of the signaling continues to grow, and with that, so does our ability to target different parts of the pathways with small molecules. Marine natural products co-evolve with their targets, and these unique molecules have complex structures with exquisite biological activities and specificities. This article offers a review of our current understanding of the signaling pathways regulating cell death, and highlights marine natural products that can affect these signaling pathways.

## 1. Introduction

The golden mean, the yin and yang, homeostasis—no matter what we call it, nature tries to maintain a balance in its functions. Thus, it is not surprising that just as proliferation is tightly regulated, regulated cell death also is. The term apoptosis was first used by Kerr, Wyllie and Currie to describe a programmed and controlled cell deletion process [1]. This early description provided us the description of some of the morphological changes that came to characterize apoptosis, such as nuclear fragmentation, the formation of apoptotic bodies, and blebbing of cell membranes. What has changed exponentially since those days is our understanding of the regulation of cell death, that those hallmarks are not present in all forms of regulated cell death, and that there are as many signaling molecules involved in driving the process as there are in stopping the process from occurring. Our knowledge of the signaling continues to grow, and with that, our ability to target different parts of the pathway with small molecules. 

Not only are there many signaling molecules involved in the regulation of the known apoptotic pathways (intrinsic and extrinsic), but our limited idea that cells died either through the controlled, regulated, energy-requiring apoptosis or the explosive inflammatory swelling known as necrosis has been changed. It turns out that many regulated processes to achieve cell death exist, including necroptosis, a regulated form of necrosis, and autophagy-dependent cell death. We also understand that apoptosis occurs beyond the intrinsic and extrinsic pathways with caspase-independent forms of apoptosis existing. Reviewing all of these pathways in detail would be the object of a much longer article and has already been done recently in a succinct and entertaining play by Dr. Douglas Green [2] and in minute detail by the Nomenclature Committee on Cell Death (NCCD) [3], to name a few recent publications. This review will revisit these pathways providing bigger strokes and highlighting the hallmarks of each kind of regulated cell death. 

All the readers are perfectly aware of how wonderful marine natural products are. Having co-evolved with their targets, these unique molecules have complex structures with exquisite biological activities and specificities. Anyone unaware of their activities has only to read Blunt’s yearly review [4] to see the many new activities and new molecules discovered recently. Therefore, it is not surprising that many natural compounds are able to regulate apoptosis, and that there are myriad pathways by which they do so. It would be impossible for a simple review to capture all marine natural products that can regulate apoptosis and there is no need to do so, as excellent recent reviews published in this journal and elsewhere have captured that. Examples include a recent review on natural products with anticancer potential [5]; an excellent, albeit slightly old, review that groups marine natural products by the pathways they activate to induce apoptosis [6]; and a more recent review that highlights selected marine natural compounds and their ability to induce apoptosis [7]. Instead, this article offers a review of our current understanding of the signaling pathways regulating cell death, and highlights selected marine natural products that can affect these signaling pathways.

## 2. Caspase Dependent Apoptosis

### 2.1. The Intrinsic Pathway of Apoptosis

Intrinsic apoptosis is described by the NCCD as a regulated cell death process that is initiated by perturbations of the intracellular or extracellular microenvironment, defined by mitochondrial outer membrane permeabilization, and executed through cysteine-aspartic protease (caspase) 3 activation [3]. This pathway can be initiated by stressful events such as growth factor withdrawal [8], changes to microtubule dynamics [9], DNA damage [10], reactive oxygen species overload [11], replication stress [12], mitotic defects [13], or endoplasmic reticulum stress [14]. These stresses lead to the critical and irreversible step in this pathway: the mitochondrial outer membrane permeabilization (MOMP), which is regulated by members of the B-cell lymphoma 2 (Bcl-2) family [2,3]. 

The Bcl-2 family is characterized as having Bcl-2 homology (BH) domains that allow them to interact with each other. Bcl-2 associated X protein (Bax) and Bcl-2 antagonist/killer 1 protein (Bak), the main pro-apoptotic members of this family that contain four BH domains, have the ability to create pores in the outer mitochondrial membrane (OMM), allowing the release of cytochrome C and the second mitochondria-derived activator of caspases/direct inhibitor of apoptosis (IAP) binding protein with low isoelectric point (Smac/Diablo) [2,3]. Bcl-2 associated agonist of cell death (Bad), Bcl-2 modifying factor (Bmf), and Bcl-2 interacting protein (Hrk) are pro-apoptotic BH3-only Bcl-2 family members that form heterodimers with anti-apoptotic members to promote apoptosis [2,3]. Bcl-2 family apoptosis regulator (Bok) is a pro-apoptotic member of the Bcl-2 family which also has the ability to form pores; however, it resides on the endoplasmic reticulum (ER) and appears to be regulated at the level of the ER and not the mitochondria [3]. However, Bok can cause MOMP in the absence of Bak or Bax. The members of the Bcl-2 family BH3 interacting domain death agonist (Bid), Bcl-2 interacting mediator of cell death (Bim), p53 upregulated modulator of apoptosis (Puma), and phorbol-12-myristate-13-acetate-induced protein 1 (Noxa) contain only the BH3 domain, and by interacting with both pro- and anti- apoptotic members of the Bcl-2 family through dimerization, can regulate their functions [2,3]. Bcl-2, B-cell lymphoma–extra large (Bcl-XL), induced myeloid leukemia cell differentiation protein (Mcl-1), Bcl-2-like 2 protein (Bcl-W), and Bcl-2-related protein A1 (A1; Bfl-1) are anti-apoptotic members of the Bcl-2 family, and contain all four BH domains. While both Bcl-2 and Bcl-XL prevent apoptosis from occurring, Bcl-XL can specifically bind to both cytochrome C and to the apoptotic peptidase activating factor 1 (Apaf-1). Bfl-1 is transcribed by the nuclear factor kappa-light-chain-enhancer of activated B cells (NFκB) in response to inflammation, reduces the release of cytochrome c from mitochondria, and blocks caspase activation [2,3]. Both pro- and anti-apoptotic members of the Bcl-2 family are further regulated through proteasomal degradation, phosphorylation, and subcellular localization [3].

The following is the current understanding of how the intrinsic pathway works: Puma, Noxa, and Bim become activated by transcriptional regulation while Bid undergoes post-translational activation. These activated pro-apoptotic BH3-only proteins interact with Bax and Bak, allowing them to oligomerize and form pores that allow the release of the BH3 only proteins from the mitochondrial outer membrane. If Bcl-2, Bcl-XL, Mcl-1, Bcl-W, or A1 (anti-apoptotic members) do not antagonize the oligomerization, then mitochondrial permeability is altered, MOMP occurs, and cytochrome C and Smac/Diablo are released [3,15]. Cytochrome C binds Apaf-1, causing a conformational change that allows the binding of caspase 9, creating the complex known as the apoptosome. Caspase 9 becomes activated through autocatalysis and formation of homodimers and heterodimers with Apaf-1 through their caspase recruitment domain (CARD) [3,15]. Caspase 9 cleaves and activates caspase 3 and caspase 7. Smac/Diablo facilitates apoptosis by binding members of the inhibitors of apoptosis (IAP) family, which include: X-linked inhibitor of apoptosis protein (XIAP), cellular inhibitor of apoptosis protein 1 (c-IAP1), baculoviral IAP repeat-containing protein 3 (c-IAP2), neuronal apoptosis inhibitor protein (NAIP), baculoviral IAP repeat-containing protein 7 (livin), and baculoviral IAP repeat-containing protein 5 (surviving). X-IAP, the best characterized member of the family, can bind caspases 3, 7, and 9. c-IAP1 and c-IAP2 inhibit apoptosis through their upregulation of cellular FADD-like IL-1β-converting enzyme (FLICE) inhibitory protein (c-Flip), inactivating caspases through their E3 ubiquitin ligase activity, activating NFkB, or promoting Smac/Diablo degradation [3,15]. Survivin can bind activated caspase 3 and caspase 7, and also plays an important role in cell cycle progression. Caspase 3 cleavage (activation) leads to DNA fragmentation, phosphatidylserine exposure, and the formation of apoptotic bodies [3]. Caspase 3 and 7, known as the executioner caspases, facilitate cell death, although they are not essential for cell death to occur [16].

#### 2.1.1. Anoikis

Anoikis is a form of intrinsic apoptosis that results from the loss of integrin-dependent attachment to the extracellular matrix [2,3]. This loss is thought to lead to MOMP through activation of the BH3-only members of the Bcl-2 family Bim and Bmf. This process is negatively regulated by activation of the mitogen-activated protein kinase 1 (Erk2), and by the anti-apoptotic members of the Bcl-2 family Mcl-1 and Bcl-2 [3]. Anoikis involves the activation of caspase 3 [3].

#### 2.1.2. Mitotic Death

Mitotic death is a variant of regulated cell death that is driven by mitotic catastrophe [3]. Mitotic catastrophe does not always end up in cell death, but can also lead to cellular senescence [3]. Mitotic catastrophe occurs when a cell cannot complete mitosis due to DNA damage accumulation, issues with mitotic machinery, or failed mitotic checkpoints [17]. Morphological characteristics of this type of regulated cell death include multinucleation and macronucleation [17]. The signaling is thought to involve p53 and subsequent activation of caspase 2 [18]. It can be regulated by the Bcl-2 family and appears to be defined by MOMP [18] in the majority of cases. In some cases, mitotic catastrophe leads to a slippage off cell cycle arrest that is driven by the degradation of cyclin B1 [19]. 

### 2.2. The Extrinsic Pathway of Apoptosis

Extrinsic apoptosis is described by the NCCD as a regulated cell death process that is initiated by perturbations of the extracellular microenvironment that are detected by cell surface receptors, involve the activation of caspase 8, which can in some cases also activate the intrinsic pathway, and is executed by caspase 3 [3]. Extrinsic apoptosis is driven by cell surface receptors: either death receptors such as Fas cell surface death receptor (Fas, CD95, APO-1, TNFRSF6), the tumor necrosis factor receptor 1 (TNFR1), and death receptors 4 and 5 (DR4 and DR5), or by dependence receptors, such as the netrin 1 receptors deleted in colorectal carcinoma (DCC) and UNC-5, the neurotrophic receptor tyrosine kinase 3 (NTRK3), and the sonic the hedgehog receptor patched 1 (PTCH1), whose activation depends on the levels of their specific ligand [3].

Regulated cell death initiated by death receptor binding by their respective ligands—Fas ligand (FasL) for Fas and TNFα for TNFR1, and tumor necrosis factor-related apoptosis-inducing ligand (TRAIL) for DR4 and DR5—leads to the formation of a death inducing signaling complex (DISC), which regulates the activation of caspase 8, and in some cases caspase 10 [3]. Although caspase 10 shares some substrate specificity with caspase 8 [20], its role is not completely understood: it is thought to promote apoptosis of primary T cells [21], but can promote cell survival in other cases [22]. It is important to note that the binding of a death receptor by its ligand does not always lead to apoptosis: in some cases the binding promotes cell proliferation and/or inflammation [23,24,25,26]. 

Binding of Fas by a Fas ligand stabilizes the preformed Fas homotrimer and induces a conformational change that allows the death domain in the Fas receptor to associate with the death domain in the Fas-associated protein with the death domain (FADD) [2,3]. FADD can in turn interact through its death effector domain (DED) with the DED of caspase 8 and/or the caspase 8 inhibitory protein Flip [3]. These interactions lead to the formation of the death inducing signaling complex (DISC), which can activate caspase 8 [3]. Binding of the TNFR1 by TNFα can lead to the formation of different signaling complexes that define different cell fates [26]. The binding of TNFR1 by TNFα activates it, and its death domain at the cytoplasmic tail rapidly recruits the adaptor protein TNF receptor associated-protein with death domain (TRADD). TRADD, in turn, recruits TNF receptor associated protein 2 (TRAF2), TRAF5, receptor associated protein kinase 1 (RIPK1), the linear ubiquitin chain assembly complex (LUBAC), and cellular inhibitor of apoptosis proteins cIAP1 and cIAP2 to form a signaling complex referred as complex I [3,26]. Complex I internalizes and converts into a death-inducing complex, complex II, with the additional recruitment of FADD and procaspase-8 [26]. Complex II can activate caspase 8 [26]. The binding of caspase 8 to FADD at the DISC leads to the homodimerization and activation through autoproteolytic cleavage of caspase 8. FLIP, a closely related protein to caspase 8 that is catalytically inactive, can inhibit caspase 8 oligomerization [3]. Caspase 8 can also be regulated through its phosphorylation or deubiquitination [3]. 

In type I cells, thymocytes and mature lymphocytes, activation of caspase 8 results in the activation of caspase 3 and caspase 7. This form of apoptosis is not regulated by members of the Bcl-2 family [3]. In type II cells—most cancer cells, hepatocytes, and pancreatic β cells, for example—caspase 3 and 7 activation by caspase 8 is regulated by XIAP and requires the cleavage of Bid. The truncated Bid (tBid) translocates to the outer mitochondrial membrane (OMM). Once at the OMM, tBid can promote regulated cell death by engaging Bax/Bak, which causes MOMP and subsequent caspase 9 activation [3]. Just like in intrinsic apoptosis, caspase 3 cleavage (activation) leads to DNA fragmentation, phosphatidylserine exposure, and the formation of apoptotic bodies [3]. 

**Figure 1:**
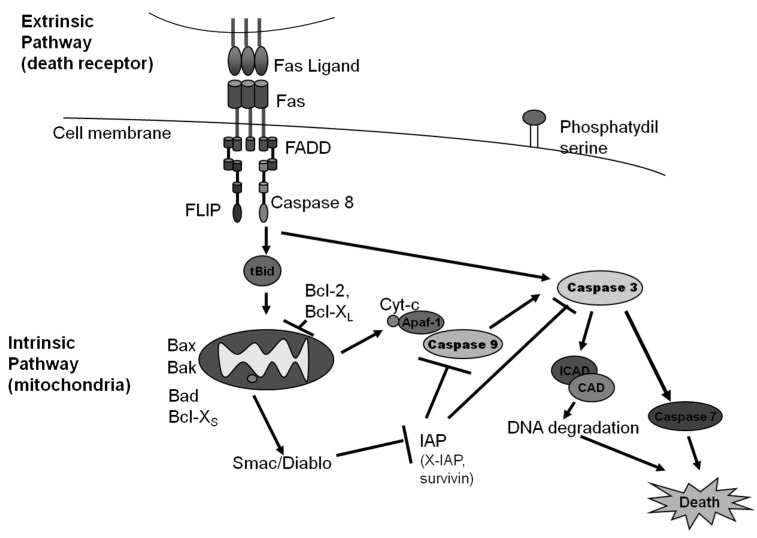
An illustration of intrinsic and extrinsic apoptosis.

As stated before, binding of a death receptor does not always lead to cell death. In particular, TNFR1 activation has many outcomes depending on the posttranslational modifications of complex I member RIPK1: polyubiquitination of RIPK1 leads to cell survival and inflammation [27]; RIPK1 phosphorylation prevents its interaction with FADD leading to RIPK1 independent apoptosis [28]; while RIPK1 deubiquitination favors it release from complex I and its association with complex II to lead to apoptosis [29]. Binding of death receptors can also lead to NF-κB activation, which leads to cell survival and inflammation [3]. An illustration of the intrinsic and extrinsic pathways of apoptosis is shown in Figure 1.

#### Dependence Receptor Driven Extrinsic Apoptosis

Dependence receptors received their name as they depend on the presence of their ligand to signal survival: the absence of their ligand can trigger apoptosis, while ligand binding signals survival [30]. Trophic ligands are molecules whose protein binding can stimulate cell growth, differentiation, or survival [31,32]. About 20 dependence receptors capable of initiating the extrinsic pathway of apoptosis have been identified to date [3,30]. These include some integrins; the neurotrophin receptors: p75 neurotrophin receptor (p75NTR), tropomyosin receptor kinase A (TrkA), tropomyosin receptor kinase C (TrkC), and rearranged during transfection (RET); the netrin-1 receptors: DCC and Unc-5 homologue 1–4 (UNC5H1-4); Kremen-1; ephrin type-A receptor 4 (EPHA4); tyrosine-protein kinase Met (MET); Anaplastic lymphoma kinase (ALK); neogenin; Plexin D1; the insulin receptor (IR); the insulin-like growth factor 1 receptor (IGF-1r); and the Sonic Hedgehog receptors: Patched (Ptc) and cell adhesion molecule-related/down-regulated by oncogenes (CDON) [30]. 

The pathways by which these molecules induce initiate extrinsic apoptosis have not been completely elucidated and appear to vary depending on which receptor initiates it [3]. 

### 2.3. Marine Natural Products That Target the Intrinsic and Extrinsic Pathways of Apoptosis

Many marine natural compounds affect microtubule dynamics (recently reviewed in Reference [33]) and can induce apoptosis as a result; examples include discodermolide [34] and leiodermatolide [35]. Discodermolide can also initiate caspase-independent cell death thought to be initiated by mitotic catastrophe in non-small cell lung cancer cells [36].

Many marine natural compounds are known to affect histone deacetylases (HDAC) [37,38,39], with examples of them inducing apoptosis being largazole [40] and psammaplin A [41]. Variolin and deoxy-variolin B induce apoptosis through their ability to inhibit cyclin dependent kinases [42]. Cephalostatin 1 has been reported to induce endoplasmic reticulum mediated cell death that does not involve caspase 8 or cytochrome c release [43], but possibly involves its release of Smac/Diablo from the mitochondria [44]. Similarly, Aurilide induces apoptosis by activating the mitochondrial dynamin like GTPase optic atrophy 1 (OPA1), which regulates the release of cytochrome C and SMAC/Diablo from the mitochondria, through its binding of prohibitin 1 [45]. 

The tight orchestration of the Bcl-2 family members to regulate apoptosis shows their importance for this pathway. Small molecules capable of acting as BH3-only proteins that can induce apoptosis by binding to pro-apoptotic members of the family, known as BH3 mimetics, appear to have a strong future as therapeutics with many in clinical trials [46]. Marinopyrrole A (Maritoclax) is a natural product derived from marine streptomycetes that appears to specifically bind Mcl-1 and target it for proteosomal degradation [47]. Some synthetic analogues of the marine natural compound jahanyne, isolated from *Lyngbya* sp., were shown to induce apoptosis in cancer cells by binding Bcl-2 [48]. The marine natural compound renieramycin M has been shown to downregulate Mcl-1 and Bcl-2 expression in a p53 dependent manner in cancer cells and sensitize them to undergo anoikis [49]. The marine natural compound spongistatin 1 has also been reported to induce anoikis in cancer cells [50].

Many marine natural compounds have been reported to inhibit NFκB [51], a molecule that transcriptionally regulates the expression of many anti-apoptotic members of the Bcl-2 family. Some of the most potent are salinosporamide A, a marine-derived bacterial proteasome inhibitor with an IC_50_ for NFκB inhibition of 11 nM [52] and the bengamides, compounds isolated from both a terrestrial bacterium *Myxococcus virescens* and the marine sponge *Jaspis coriacea* with an IC_50_ for NFκB inhibition around 80–90 nM [53]. Other marine natural compounds, such as spongiatriol and microsclerodermin A, exhibit low micromolar activity (IC_50_ for NFκB inhibition of 3.4 and 2.4 μM, respectively) and induce apoptosis in cancer cells with constitutive NFκB activation [54,55]. 

**Figure 2:**
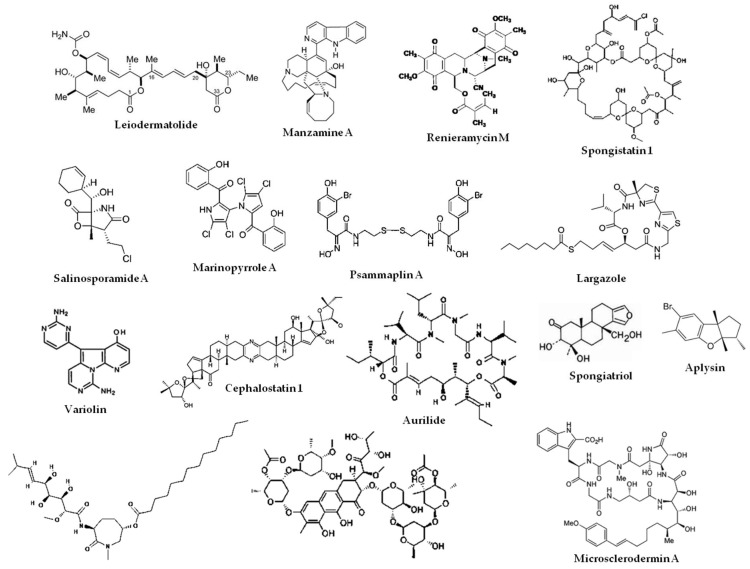
Structures of some marine natural products that target intrinsic and extrinsic apoptosis.

Many marine natural products help cancer cells overcome their resistance to the death receptor ligand TRAIL with examples including manzamine A [56], chromomycins A2 and A3 [57], and aplysin [58]. Many natural compounds have been shown to exhibit neurotrophic activities; among marine natural products, manzamine A was reported to have this activity [32]. Finally, many marine natural compounds with potent cytotoxic activity exert their activity through induction of apoptosis (reviewed in References [5,7]). The structures of some of these marine natural products that target apoptosis are shown in Figure 2.

## 3. Caspase-Independent Regulated Cell Death

### Paraptosis

Paraptosis is a form of regulated cell death without the typical hallmarks of apoptosis such as chromatin condensation, DNA fragmentation, or caspase activation [59]. The hallmark for this type of regulated cell death is cytoplasmic vacuolation accompanied by swelling of the mitochondria and the endoplasmic reticulum (ER) [59]. Paraptosis can be initiated by potassium channel activation [60], the TNF family receptor TAJ/TROY, and the IGFR1 [59]. Signaling is thought to be mediated by mitogen-activated protein kinases and to be able to be inhibited by ALG-2-interacting protein X (AIP/Alix) [59]. ALG-2 is a calcium binding protein associated with cell death.

Yessotoxin, a toxin from a dinoflagellate, has been shown to induce paraptosis in a murine muscle cell line [61]. Two acetylene alcohols from the sponge *Cribrochalina vasculum* were found to inhibit phosphorylation of, and signaling by, IGFR1 [62].

## 4. Regulated Necrosis

### 4.1. Necroptosis

Necroptosis is a form of regulated cell death that is initiated by changes to cellular homeostasis that depends on the mixed lineage kinase like (MLKL), the receptor interacting protein kinase 3 (RIPK3), and in some cases, on the kinase activity of the receptor interacting protein kinase 1 (RIPK1) [3].

Necroptosis can be initiated by death receptors such as Fas, but is mainly activated by TNFR1, toll-like receptors (TLR) TLR3 and TLR4, and by the Z-DNA binding protein 1 (ZBP1) initiating a cell cascade that exhibits necrosis like morphology [3]. Necroptosis is initiated by TNFR1, which activates RIPK1. Active RIPK1 subsequently activates RIPK3 if caspase 8 is not active [3]. The toll-like receptors (TLR) can also activate RIPK3 [63]. Once activated, RIPK3 phosphorylates MLKL, which oligomerizes and translocates to the plasma membrane where they bind phosphatidyl inositol phosphate and trigger plasma membrane permeabilization [3]. Caspase 8, in conjunction with FADD and FLIP, negatively regulate necroptosis [64,65]. In some cases, RIPK1 can inhibit RIPK3-driven necroptosis and caspase 8 induced apoptosis [66]. RIPK1 can also activate NFκB [67]. 

Many of the components of necroptosis signaling, including ZBP1, RIPK3, MLKL, and TNFR1 are also important in the regulation of the inflammasome [3]. The inflammasome activates caspase 1, which can lead to the secretion of interleukins 1 and 18, but can also lead to the cleavage and activation of Bid, and caspases 3 and 7 to promote apoptosis [15].

### 4.2. Ferroptosis

Ferroptosis is a form of regulated cell death that is initiated by oxidative changes in the microenvironment, that is under regulation by glutathione peroxidase 4 (GPX4), and inhibited by lipophilic antioxidants and iron chelators [3].

This form of regulated cell death is driven by the toxic accumulation of lipid hydroperoxides and has a necrotic morphology [68]. Ferroptosis is driven by the loss of activity of the lipid repair enzyme GPX4, which is followed by an accumulation of lipid-based ROS [68]. Iron is required for ferroptosis and catalyzes the conversion of peroxides into free radicals [68]. Antioxidants that act as ROS scavengers can inhibit ferroptosis [3].

### 4.3. Pyroptosis

Pyroptosis is a form of regulated cell death that relies on the gasdermins creating pores in the plasma membrane usually as the result of caspase activation by inflammation [3]. Pyroptosis is characterized by chromatin condensation (different from the one that occurs with apoptosis), and is driven by activation of one or more caspases, including caspases 1, 4, 5, and 11 [69]. Caspase 3 has also been shown to activate pyroptosis in some cases [70]. Intracellular lipopolysaccharides are sensed by caspases 4, 5, and 11 [69]. Caspase 1 is activated by inflammasomes [69]. Once activated, the caspases cleave gasdermin D (GSDMD) [3]. GSDMD constitutes a large gasdermin family that binds lipids and has the ability to form pores in the plasma membrane as a response to microbial infection and other danger signals [69]. Pyroptosis is commonly accompanied by the secretion of IL-1β and IL-18 secretion, and thus, of inflammation [3]. Gasdermins A, B, C, and E have also been shown to have the ability to form pores in the membrane and activate pyroptosis [3]. Caspase 3 has been shown to cleave GSDME to initiate pyroptosis [70].

### 4.4. Parthanatos

Parthanatos is a form of regulated cell death initiated by poly(ADP-ribose) polymerase 1 (PARP1) hyperactivation as a result of DNA damage, oxidative stress, hypoglycemia, hypoxia, or inflammation that is caused by bioenergetic collapse coupled by DNA fragmentation mediated by both the apoptosis inducing factor (AIF) and the macrophage migration inhibitory factor (MIF) [3]. 

DNA damage or stress signals lead to the formation of reactive nitrogen species which lead to PARP1 hyperactivation. This hyperactivation in turn causes nicotinamide adenine dinucleotide (NAD+) and ATP depletion and redox collapse leading to an accumulation of poly(ADP-ribose) polymers and poly(ADP-ribosyl)ated proteins at the mitochondrial leading to loss of mitochondrial membrane potential and MOMP [3,71]. Poly(ADP-ribose) polymers can directly bind AIF, causing it to be released from the mitochondria and translocate to the nucleus [3,71]. In the cytosol, AIF binds MIF, promoting MIF’s translocation to the nucleus, where it catalyzes DNA cleavage [3]. AIF in the nucleus promotes DNA fragmentation and chromatin condensation [3,71]. Poly(ADP-ribose) polymers can also bind the hexokinase 1 (HK1) leading to inhibition of glycolysis and causing a bioenergetic collapse that leads to regulated cell death [3]. 

### 4.5. Mitochondrial Permeability Transition (MPT)-Driven Necrosis

MPT-driven necrosis is a type of regulated cell death that is triggered by changes to the intracellular microenvironment, including severe oxidative stress and cytosolic Ca^+2^ overload that relies on cyclophilin D (CYPD) [3]. This type of necrosis occurs when the permeability transition pore complex (PTPC), a supramolecular complex assembled at the junction between the inner and outer mitochondrial membranes, is opened. The proteins that compose and regulate the PTPC are still under investigation [72]. MPT-driven necrosis is regulated by members of the Bcl-2 family, the dynamin-related protein 1 (DRP1), and p53 [3]. Mitochondrial calcium overload may also trigger this form of regulated necrosis [3].

### 4.6. Marine Natural Products That Target Regulated Necrosis

**Figure 3:**
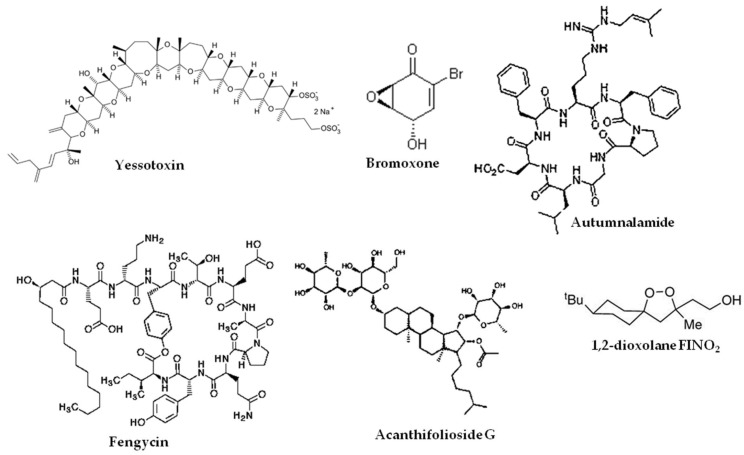
Structures of some marine natural products that target regulated necrosis.

The marine natural product bromoxone has been reported to inhibit caspase 1 [73]. Autumnalamide can affect calcium fluxes and binds cyclophilin D [74]. The fengycins kill plant fungi by inducing ROS, PARP cleavage, and chromatin condensation [75]. Acanthifolioside G exhibits anti-oxidant activity and increases glutathione peroxidase activity in human U373 astrocytoma cells [76]. The 1,2-dioxolane FINO_2_, an analogue of the natural plakinic acids, was shown to induce ferroptosis and inhibit GPX4 in HT-1080 fibrosarcoma cells [77]. The β-carboline RSL3 compound induces ferroptosis in cells that have oncogenic RAS [78]. The structures of some marine natural compounds that target regulated necrosis are shown in Figure 3.

## 5. Autophagy-Dependent Cell Death

Autophagy-dependent cell death is a form of regulated cell death that depends on the autophagic machinery [3]. Autophagy involves the sequestration of organelles into vesicles followed by the degradation of those organelles by lysosomes after formation of the autophagosome [59]. Autophagy is started as a response to stress, and thus it mainly has cytoprotective effects [3]. However, in certain cases, its activation results in cell death [3]. Beclin1, an important mediator of autophagy, can induce and regulate autophagy and membrane trafficking in several physiological and pathological processes, but can also regulate apoptosis [79,80]. Beclin 1 in turn can be regulated by Bcl-XL, Bim, and FLIP [81]. Autophagy is activated alongside other forms of cell death, such as Fas-mediated extrinsic apoptosis, ferroptosis, and necroptosis, where autophagy facilitates those forms of cell death rather than mediates cell death itself [3]. Nevertheless, autophagy-mediated cell death exists; in *Drosophila*, autophagic cell death drives the disposal of larval tissues [82,83]. This cell death appears to be regulated by ubiquitin activating enzyme 1 (Uba1) and to be independent of the autophagy related (Atg) proteins 7 and 3 [3]; meanwhile, these Atgs are essential for starvation-induced autophagy [3]. On the other hand, overexpression of Atg1 is sufficient to induce this autophagic cell death [15]. In Bax- and Bak-deficient mice, autophagic cell death is driven by Beclin 1 and Atg 5 and 7 [81]. Similarly, autophagic cell death was shown to be driven by oncogenic Ras inducing the expression of Noxa and Beclin-1 in ovarian cancer cells [84]. Autosis is a type of autophagy-dependent cell death that is regulated by the plasma membrane Na^+^/K^+^-ATPase [85].

Autophagic cell death is not a common occurrence. While many marine natural compounds can affect autophagy either inducing it (chromomycin A2, psammaplin A, ilimaquinone [86], coibamide A, and apratoxin A [87]), or inhibiting it (manzamine A [88] and scalarin [89]), to our knowledge, only yessotoxin has been reported to induce autophagic cell death in glioma cell lines [90].

## 6. Conclusion

As our understanding of the pathways regulating cell death and cell fate increases, so do the options of finding marine natural products that may modulate their activity. Table 1 shows some of the pathways discussed along with their triggers and marine natural products known to target that pathway.

Many of the signaling pathways overlap and depend on the cell type, the damage triggering the regulated cell death, and the capacity of the cell to repair damage, and determines which form of regulated cell death occurs. The existence of this variety of pathways, as well as the ability of cells to recover from many of these events, makes it imperative to use the correct assays that distinguish the different forms of regulated cell death. 

The ability of marine compounds to affect regulated cell death pathways makes them potential anti-cancer treatments, and some, if not all, were discovered as such. For example, leiodermatolide shows selectivity for cancer cells over normal cells [35,91], has anti-tumor properties [35], and is the subject of an elegant synthesis [92] to address the common issue of obtaining more compound that plagues marine natural compounds. Two different recent reviews highlight the potential as anti-cancer agents of many of these compounds [5,7].

## Figures and Tables

**Table 1:** Pathways of regulated cell death, their initiating events, and marine natural compounds that can affect these pathways.

Regulated Cell Death Pathway	Extrinsic Apoptosis	Intrinsic Apoptosis	Anoikis	Paraptosis	Necroptosis	Autophagic Cell Death
Initiated by:	Death receptors Dependence receptors	Changes to microtubule dynamics Growth factor withdrawal DNA damage ROS overload Cellular stress	Loss of integrin-dependent attachment to the extracellular matrix	Potassium channel activation TAJ/TROY IGFR1	TNFR1 TLR3 TLR4 ZBP1	Beclin-1 Atg-1 Atg-5 Atg-7 Noxa
Marine natural products that target this pathway:	Leiodermatolide Manzamine A	Largazole Cephalostatin 1 Marinopyrrole A Renieramycin A	Renieramycin M	Yessotoxin	Bromoxone	Yessotoxin
